# Identification of lncRNAs Involved in PCV2 Infection of PK-15 Cells

**DOI:** 10.3390/pathogens9060479

**Published:** 2020-06-17

**Authors:** Jin He, Chaoliang Leng, Jiazhen Pan, Aoqi Li, Hua Zhang, Feng Cong, Huanan Wang

**Affiliations:** 1 College of Animal Sciences, Zhejiang University, Hangzhou 310058, China; hejin@zju.edu.cn (J.H.); 17816899089@zju.edu.cn (J.P.); 119989@zju.edu.cn (A.L.); 3160100304@zju.edu.cn (H.Z.); 2 Henan Provincial Engineering and Technology Center of Animal Disease Diagnosis and Integrated Control, Henan Key Laboratory of Insect Biology in Funiu Mountain, Nanyang Normal University, 1638 Wolong Road, Wolong District, Nanyang 473061, China; lenghan1223@nynu.edu.cn; 3 Guangdong Provincial Key Laboratory of Laboratory Animals, Guangdong Laboratory Animals Monitoring Institute, Guangzhou 510640, China; fcong@gdlami.com

**Keywords:** PCV2, lncRNA, pig, RNA-seq

## Abstract

Porcine circovirus type 2 (PCV2) can cause severe disease in infected pigs, resulting in massive economic loss for the swine industry. Transcriptomic and proteomic approaches have been widely employed to identify the underlying molecular mechanisms of the PCV2 infection. Numerous differentially expressed mRNAs, miRNAs, and proteins, together with their associated signaling pathways, have been identified during PCV2 infection, paving the way for analysis of their biological functions. Long noncoding RNAs (lncRNAs) are important regulators of multiple biological processes. However, little is known regarding their role in the PCV2 infection. Hence, in our study, RNA-seq was performed by infecting PK-15 cells with PCV2. Analysis of the differentially expressed genes (DEGs) suggested that the cytoskeleton, apoptosis, cell division, and protein phosphorylation were significantly disturbed. Then, using stringent parameters, six lncRNAs were identified. Additionally, potential targets of the lncRNAs were predicted using both cis- and trans-prediction methods. Interestingly, we found that the *HOXB* (Homeobox *B*) gene cluster was probably the target of the lncRNA *LOC106505099*. Enrichment analysis of the target genes showed that numerous developmental processes were altered during PCV2 infection. Therefore, our study revealed that lncRNAs might affect porcine embryonic development through the regulation of the *HOXB* genes.

## 1. Introduction

Porcine circovirus (PCV), which belongs to the family Circoviridae, is a nonenveloped virus with a single-stranded circular DNA [1]. Currently, there are three known types of PCVs. PCV1, which was first identified in the pig kidney cell line, is nonpathogenic to pigs [2,3]. PCV3 is a newly identified pathogen with the first report in North America. The afflicted pigs have symptoms of porcine dermatitis and nephropathy syndrome and reproductive problems [4]. Another PCV is porcine circovirus type 2 (PCV2), which is the primary threat to the global swine industry leading to massive economic loss annually [5]. PCV2 is the cause of pig postweaning multisystemic wasting syndrome (PMWS), which is characterized by progressive loss of weight, respiratory difficulty, jaundice, and reproductive failure [6]. Microscopically, PMWS is associated with severe lymphocyte depletion and multiple organ inflammation [7,8]. Thus, the study of PCV2 is of great importance. PCV2 has a genome size of 1.7 kb with at least three open reading frames (ORFs). ORF1 encodes the proteins Rep and Rep’, both of which are essential for virus replication [9]. ORFs 2 and 3 reside in the opposite strand, encoding the pathogenic structure protein capsid and the peptide involved in the host cell apoptosis, respectively [10,11].

Long noncoding RNAs (lncRNAs) are a group of noncoding RNAs that are longer than 200 nt. Except for their small coding potential, lncRNAs are similar to ordinary mRNAs in terms of exon-intron organization, 5′ cap, and 3′ polyadenine tracts [12,13]. Additionally, lncRNAs can be transcribed from intronic or intergenic regions by the RNA polymerase II in a sense and antisense manner [14]. Previous studies show that lncRNAs have poor sequence conservation, so they are species-specific [15]. However, accumulating evidence suggests that structural information, functions, and synteny are also constituents of the conservation of lncRNAs [16,17]. Since their discovery, lncRNAs have been implicated in numerous biological processes, including cell signaling [18], epigenetic regulation [19], enhancer trapping [20], human diseases [21,22], and virus-host interactions [23,24]. However, until recently, only one study regarding the effect of lncRNAs on PCV2 infection has been published, in which the lncRNA-regulated mRNAs were found to be enriched in the DNA/RNA binding, transcription factor activity, immune response, the MAPK (mitogen-activated protein kinase) signaling pathway, and cytokine–cytokine receptor interaction [25].

In our study, using more stringent screening parameters than were previously used, we identified six differentially expressed lncRNAs upon PCV2 infection. Enrichment analysis of the potential targets of the lncRNAs suggested that developmental processes might be disturbed. Therefore, our study provides novel insight into lncRNAs in PCV2 infection.

## 2. Materials and Methods

### 2.1. Cell Culture and PCV2 Infection

PK-15 cells free of PCV2 were purchased from the American Type Culture Collection (ATCC, Manassas, VA, USA), and they were cultured in Dulbecco’s modified Eagle medium (HyClone, GE, Beijing, China) supplemented with 10% fetal bovine serum (HyClone, GE, Beijing, China). The cell line was used because it is a widely accepted in vitro infection model, especially in the field of PCV2 virology, from which underlying molecular and cellular mechanisms of PCV2 infection have been learned [26,27,28,29,30,31]. The PCV2 strain 17HNA1 (Accession no.: MT423827) was used in our study. The infection procedures strictly followed a previously published protocol [32] with minor modifications: multiplicity of infection (MOI) was set at 0.1, and the PK-15 cells were harvested at 72 h postinfection. Viral and mock infections were repeated three times. Therefore, the mock infections were named as con-1, con-2, and con-3, while the PCV2 infection repeats were designated as PCV2-1, PCV2-2, and PCV2-3.

### 2.2. RNA Preparation, Quantitative Real-Time PCR (qRT-PCR), and RNA-Seq

Seventy-two hours postinfection, the PCV2-infected and mock-infected cells were washed with ice-cold PBS (Sigma Aldrich, Shanghai, China) three times before being subjected to total RNA extraction using the Mini Best Universal RNA Extraction kit (Takara, Dalian, China). Next, the extracted RNA was tested using the Agilent 2200 Analyzer for quality control (QC), and those samples with an RNA Integrity Number (RIN) greater than 8 passed the QC process. Then, the RNA was subjected to RNA-seq using the Illumina HiSeq X Ten platform (San Diego, CA, USA) according to the pipeline of Novel Bioinformatics Inc. (Shanghai, China). The raw sequencing data were filtered using the fastp [33] to obtain clean reads, which were subsequently mapped to the porcine genome using the Hisat2 [34]. Then, the raw counts were obtained using the FeatureCounts [35]. Next, differentially expressed transcripts were calculated based on the following parameters: |log_2_fold change| > 0.2630 (fold change greater than 1.2 or lesser than 5/6) and adjusted *p*-value (false discovery rate, FDR) < 0.05 using the DESeq2 tool [36]. The identification of lncRNAs used a strategy described in a previous report [25]. The sequenced reads were submitted to the Gene Expression Omnibus with the accession number: GSE149711.

qRT-PCR was then carried out to confirm the expression levels of the selected transcripts, and it was performed according to a previously described protocol [37]. The transcripts tested were *ORF2*, *NGFR*, *PIGR*, *SERPINF1*, *LOC106505099*, *LOC106509609*, *TRIM29*, and *LOC100512687*, the latter two of which served as the negative controls. The *ACTB* was used as an internal control. All primers used are listed in Table S1. qRT-PCR data were analyzed using the 2^−ΔΔCt^ method [38].

### 2.3. LncRNA Target Prediction

LncRNA cis-regulated target genes were identified by searching the protein-coding genes 50 kb up or downstream of each lncRNA. Then, the expression level of each of the identified lncRNAs was compared with all the sequenced reads. Transcripts with Pearson’s correlation coefficient >0.99 and adjusted *p*-value < 0.05 were considered potential trans-targets of lncRNAs.

### 2.4. Enrichment Analysis

The differentially expressed genes (DEGs) identified by RNA-seq and the lncRNA targets were submitted to the Database for Annotation, Visualization and Integrated Discovery (DAVID) [39,40] for the identification of significantly altered Gene Ontology (GO) [41] and Kyoto Encyclopedia of Genes and Genomes (KEGG) [42] terms using the default parameters (EASE score < 0.1).

### 2.5. Statistics

Student’s t-tests were used to compare the difference in transcripts between PCV2-infected and mock-infected cells. Data are presented as the mean ± standard errors of the means (SEMs). Type I error was set at 0.05.

### 2.6. Ethical Statement

The experiment was approved by the Animal Welfare Committee of Zhejiang University (No. 11,834 issued on 26 February 2018).

## 3. Results and Discussion

**Figure 1:**
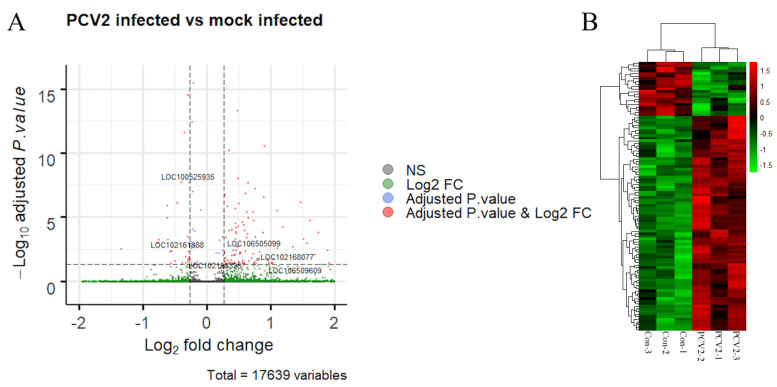
Analysis of the RNA-seq results. (**A**) The volcano plot shows the differentially expressed transcripts (red dots) between porcine circovirus type 2 (PCV2) infection versus mock infection. Each dot represents a single transcript, while the blue dots or green dots represent the transcripts that only satisfy either a log_2_fold change or an adjusted *p*-value, respectively. The six long noncoding RNAs (lncRNAs) are also labeled. (**B**) Heatmap with clustering analysis of the differentially expressed genes (DEGs). Each row indicates an individual RNA, with columns representing the samples.

Total RNA from the PK-15 cells was extracted and subjected to the RNA-seq. After the QC process and removal of ribosomal RNA sequences, an average of 84% of the sequence reads was mapped to the pig genome (Table S2), demonstrating that RNA-seq provided a reliable result. The positions of the reads were mostly located in the coding sequences or exonic regions (Figure S1). A total of 38,841 transcripts were identified in the six sequence samples, of which 111 transcripts were differentially expressed; 87 were upregulated, and 24 were downregulated (Figure 1A and Table S3). Then, a heatmap with clustering analysis using all the DEGs was constructed, in which PCV2-infected cells exhibited significantly different expression patterns compared to the mock-infected cells (Figure 1B). Within this group of transcripts, six differentially expressed lncRNAs were also observed; 4 were upregulated, and 2 were downregulated (Figure 1A and Table S4).

**Figure 2:**
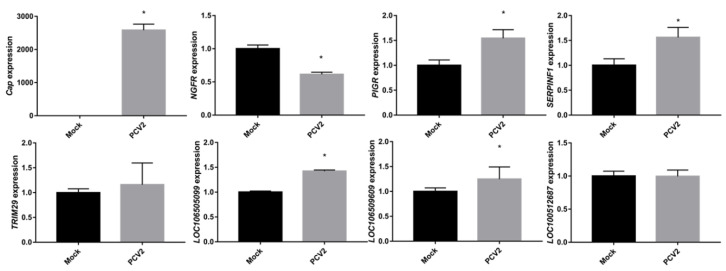
qRT-PCR validation of selected mRNAs and lncRNAs. *Cap* (*ORF2*) levels were higher in PCV2-infected cells than in mock-infected cells, demonstrating successful infection. Other genes or lncRNAs have similar patterns of expression to the RNA-seq results. Data are presented as the mean ± standard errors of the means (SEMs) with * indicating a *p*-value < 0.05.

Next, qRT-PCR was employed to confirm the expression of some selected genes and lncRNAs. The *Cap* expression level was significantly higher in the PCV2-infected groups, demonstrating the successful infection of PK-15 cells (Figure 2). Other selected genes and lncRNAs, such as *NGFR*, *PIGR*, *SERPINF1*, *LOC106509609*, and *LOC106505099*, showed expression patterns that were similar to the data obtained by RNA-seq (Figure 2 and Table S3). Accordingly, the negative controls (*TRIM29* and *LOC100512687*) had comparable expression levels in the PCV2- and mock-infected cells.

Analysis of the underlying molecular mechanisms of PCV2 infection and replication is key to the prevention of the virus. Previously, several high-throughput approaches have been employed to study mRNA, miRNA, and protein changes after PCV2 infection. In these studies, the DEGs analyzed by microarray or RNA-seq were enriched in the immune response, cytokine production, and apoptosis upon viral infection using in vivo or in vitro methods [43,44,45,46,47,48,49]. Later, miRNA was also tested for its role in the disease. Analysis of the targets of miRNAs indicated that disease resistance, MAPK signaling, and transcriptional regulation were involved [47,50]. Direct quantifications of the protein amounts after PCV2 infection revealed that there were altered levels of cytoskeletal proteins, stress response proteins, signal transduction proteins, and proteins involved in the ubiquitin-proteasome processes [32,51]. However, only one study had previously been performed to evaluate the effect of lncRNAs on PCV2 infection [25]. Fang et al. used PCV2 to infect the porcine intestinal epithelial cell line IPEC-J2 to mimic porcine enteritis. In their study, some interesting conclusions were drawn; they found that transcriptional regulation, nucleic acid binding, MAPK signaling, and cytokine-related pathways were disturbed upon PCV2 infection. However, they used the *p*-value instead of the adjusted *p*-value to identify the altered lncRNAs and mRNAs, which would substantially increase the type I error, leading to false positive findings. For example, in their lncRNA list, no lncRNA identified has an adjusted *p*-value of less than 0.05 [25]. Therefore, in the current study, we aimed to study whether some differentially expressed lncRNAs could be found using more stringent screening parameters.

Of the 111 differentially expressed transcripts, 105 were assigned to the protein-coding genes. Thus, these DEGs were analyzed using the DAVID tool for the identification of enriched biological processes (BP), cellular components (CC), and molecular functions (MF). As shown in Table 1, the DEGs were mainly involved in cell division, apoptosis, and the cytoskeleton and protein phosphorylation processes. Cell division and apoptosis have been well studied previously [52,53]. The involvement of cytoskeletal processes was also noticed in proteomic studies, indicating that the virus might affect cell division through the mitotic spindle [32,51]. The protein phosphorylation process is associated with numerous critical biological processes, such as the MAPK signaling pathway, which has been shown to be essential in viral infection [30,31].

KEGG analysis indicated that Rap1, Ras signaling pathways, and arginine biosynthesis were significantly enriched. Currently, no research has been conducted regarding the roles of arginine biosynthesis and Rap1 signaling in PCV2 infection. However, Ras is the upstream effector of MAPK signaling, which promotes infected cell proliferation [30,31]. In our enrichment analysis, no immune-related terms were found. This disparity may be because we used an MOI of 0.1, resulting in minimally infected PK-15 cells [26].

With our experimental conditions in combination with the stringent screening parameters, only six lncRNAs were found to be differentially expressed (adjusted *p*-value < 0.05, Table S4). Thus, these lncRNAs may be sensitive to PCV2 infection. Next, to functionally annotate these lncRNAs, the lncRNA targets were predicted. Currently, there are three main prediction approaches. The first one is cis-regulated target prediction, which is based on the fact that lncRNA can regulate its nearby gene transcription [54]. Thus, the genes adjacent to the lncRNA within an appropriate range are all potentially regulated. The second approach predicts the targets based on the coexpressing pattern. The underlying mechanism is that the interacting lncRNA and mRNA might have a similar expression pattern. The third method is directly computing the interaction potential of lncRNA and its targets using parameters such as free energy change, secondary structural information [55,56,57]. The former two methods can produce fast prediction results; however, the prediction is indirectly based on correlation, which needs to be validated experimentally. The computational approach can calculate the direct interaction of lncRNA and its targets. However, it also needs intensive computational facilities. After weighing the pros and cons, cis-regulated target prediction and coexpression methods were employed in our current study, and the results were then shown in Table 2. Analysis of the neighboring genes showed that 18 genes were potential targets; however, no cis-target was found for *LOC102158335*. One interesting finding is that lncRNA *LOC106505099* was located within the *HOXB* (Homeobox B) gene family, which is critical for the development of embryos [58,59,60]. Coexpression analysis using stringent parameters predicted that only three genes, *DNAAF3*, *PPP3CB*, and *CKMT1A*, were correlated with *LOC102168077*, *LOC100525935*, and *LOC102161888*, respectively. Thus, the three genes might be regulated by their respective lncRNAs in a trans manner. 

Next, to predict the potential biological roles of these lncRNAs in the infected PK-15 cells, GO analysis was performed using the potential target genes of the lncRNAs. The enrichment analysis showed that these target genes were mainly located in the nucleus (CC) and were involved in two MF processes: DNA binding and transcription factor regulation (Table 3). GO_BP analysis revealed the involvement of DNA transcription and several developmental processes, e.g., embryonic skeletal development, facial nerve structural organization, and rhombomere 4 development, suggesting the role of PCV2 in organ development. Previous studies on pig embryos showed that PCV2-exposed embryos had a higher mortality rate, especially when the zona pellucida was compromised, resulting in a low pregnancy rate of sows [61,62,63]. These results indicated that PCV2 might affect the reproductive systems through lncRNA-mediated modulation of *HOXB* genes. Virus infection mediated dysregulation of *HOX* genes has been reported before. In one study, the hepatitis B virus can induce *HOXA10* gene expression in human cells [64]. Another report showed that human papillomavirus gene expression could be modulated by *HOXD9* in cervical cancer [65]. The Epstein-Barr virus activates NKL homeobox genes in B-cell lymphoma [66]. Thus, it is reasonable to link PCV2 infection with *HOXB* gene regulation. Subsequently, we searched the human genome for the presence of possible lncRNA orthologs. Based on syntenic information, *LOC106505099* and *LOC102161888* have potential human orthologs *HOXB-AS1* and *SMIM15-AS1*, respectively. *LOC106505099* and *HOXB-AS1* are both located between *HOXB2* and *HOXB3* and are in the antisense direction, while *LOC102161888* and *SMIM15-AS1* are adjacent to the *SMIM15* gene in a tail-to-tail manner. However, only *HOXB-AS1* and *LOC106505099* showed some extent of sequence conservation according to a Basic Local Alignment Search Tool (BLAST) search of the pig transcriptome using the *HOXB-AS1* sequence (ENST00000435312.5). Functionally, some lncRNAs residing in the *HOX* gene cluster have been associated with developmental regulation through the modulation of neighboring *HOX* genes [67,68]. Moreover, two functional studies regarding *HOXB-AS1* in two cancer cell lines indicated that the lncRNA might promote proliferation and migration of cancer cells by either acting as a miRNA sponge or by participating in the Wnt/beta-catenin signaling pathways [69,70]. Another research project on mouse ortholog *Hoxb3os* showed that the lncRNA was downregulated in cystic kidneys, and regulated EIF2, mTOR, and EIF4 signaling pathways [71]. However, the direct link between *HOXB-AS1/LOC106505099* and development has yet to be established, and further functional validation is required to unveil the biological roles of *HOXB-AS1* or *LOC106505099* in porcine embryo development and PCV2 infection.

## 4. Conclusions

Using the RNA-seq technique, we identified differentially expressed lncRNAs upon PCV2 infection with stringent screening parameters. Through analysis of the targets of these lncRNAs, we found that *LOC106505099* might be involved in the stages of embryonic development through the regulation of *HOXB* genes. These findings suggest a novel association between PCV2 infection and embryonic development, which would shed new light on the pathological mechanisms of PCV2 infection.

## Figures and Tables

**Table 1:** Significantly enriched Gene Ontology (GO) and Kyoto Encyclopedia of Genes and Genomes (KEGG) terms by the Database for Annotation, Visualization and Integrated Discovery (DAVID) analysis.

Category	Term/Pathway	Count	Genes	*p*-Value
GO_BP	GO:0007051~spindle organization	4	*AURKA*, *AURKB*, *ASPM*, *RGS14*	4.78 × 10^−5^
GO_BP	GO:0007067~mitotic nuclear division	7	*TIMELESS*, *NCAPG2*, *AURKA*, *AURKB*, *ASPM*, *RGS14*, *CDCA3*	9.01 × 10^−4^
GO_BP	GO:0051301~cell division	7	*CCNB3*, *TIMELESS*, *NCAPG2*, *KIF18B*, *AURKA*, *RGS14*, *CDCA3*	0.0050
GO_BP	GO:0051973~positive regulation of telomerase activity	3	*PKIB*, *AURKB*, *KLF4*	0.0076
GO_BP	GO:0060291~long-term synaptic potentiation	3	*SYT12*, *VAMP2*, *RGS14*	0.013
GO_BP	GO:0032532~regulation of microvillus length	2	*VIL1*, *CDHR2*	0.031
GO_BP	GO:0051256~mitotic spindle midzone assembly	2	*KIF23*, *AURKB*	0.036
GO_BP	GO:0007288~sperm axoneme assembly	2	*PLA2G3*, *UBE2B*	0.036
GO_BP	GO:0043154~negative regulation of cysteine-type endopeptidase activity involved in apoptotic process	3	*VIL1*, *NGFR*, *KLF4*	0.039
GO_BP	GO:0030855~epithelial cell differentiation	3	*GSTA1*, *VIL1*, *CDHR2*	0.040
GO_BP	GO:0046777~protein autophosphorylation	4	*PAK2*, *FLT4*, *AURKA*, *AURKB*	0.0435
GO_BP	GO:1904355~positive regulation of telomere capping	2	*PKIB*, *AURKB*	0.070
GO_BP	GO:0048514~blood vessel morphogenesis	2	*SERPINF2*, *FLT4*	0.074
GO_BP	GO:0071539~protein localization to centrosome	2	*CEP131*, *AURKA*	0.078
GO_BP	GO:0042730~fibrinolysis	2	*SERPINF2*, *PROS1*	0.091
GO_BP	GO:0050769~positive regulation of neurogenesis	2	*SERPINF1*, *RGS14*	0.091
GO_BP	GO:0043171~peptide catabolic process	2	*ACY1*, *PM20D1*	0.095
GO_BP	GO:0090316~positive regulation of intracellular protein transport	2	*CEP131*, *VAMP2*	0.095
GO_BP	GO:0048754~branching morphogenesis of an epithelial tube	2	*MKS1*, *TIMELESS*	0.099
GO_CC	GO:0005819~spindle	5	*KIF23*, *FAM96B*, *AURKA*, *AURKB*, *RGS14*	0.0020
GO_CC	GO:0045171~intercellular bridge	3	*KIF23*, *CEP131*, *AURKB*	0.016
GO_CC	GO:0005886~plasma membrane	28	*CTNNAL1*, *SLC2A11*, *ADCY7*, *ATP10D*, *SLC19A2*, *MAP3K7*, *NRCAM*, *PAK2*, *MLKL*, *SLC8B1*, *FAM127A*, *ANO9*, *FLT4*, *PIK3C2B*, *SYT12*, *VIL1*, *LRRC45*, *UBE2B*, *RGS14*, *STAT2*, *AJUBA*, *ANKRD13B*, *CDH16*, *CD82*, *NGFR*, *VAMP2*, *PLA2G3*, *PROS1*	0.018
GO_CC	GO:0030496~midbody	4	*KIF23*, *AURKA*, *AURKB*, *ASPM*	0.019
GO_CC	GO:0032133~chromosome passenger complex	2	*AURKA*, *AURKB*	0.022
GO_CC	GO:0045111~intermediate filament cytoskeleton	3	*DDX60*, *DST*, *IP6K2*	0.022
GO_CC	GO:0015630~microtubule cytoskeleton	4	*CEP131*, *TIMELESS*, *AURKA*, *DST*	0.023
GO_CC	GO:0005829~cytosol	23	*GSTA1*, *KIF23*, *CTNNAL1*, *SCPEP1*, *CEP131*, *ACY1*, *NOS1AP*, *PIK3C2B*, *AURKA*, *SELENBP1*, *AURKB*, *STAT2*, *MAP3K7*, *AJUBA*, *MKS1*, *RNF115*, *PAK2*, *MLKL*, *VAMP2*, *NGFR*, *DST*, *KPNA2*, *CDCA3*	0.029
GO_CC	GO:0043203~axon hillock	2	*SERPINF1*, *AURKA*	0.031
GO_CC	GO:0005654~nucleoplasm	20	*KIF23*, *FAM96B*, *TONSL*, *VIL1*, *LRRC45*, *AURKA*, *ATP10D*, *AURKB*, *RBBP7*, *UBE2B*, *STAT2*, *CCNB3*, *SUGP2*, *PAK2*, *TIMELESS*, *NCAPG2*, *NGFR*, *KPNA2*, *KLF4*, *IP6K2*	0.034
GO_CC	GO:0072687~meiotic spindle	2	*AURKA*, *ASPM*	0.039
GO_CC	GO:0005813~centrosome	6	*KIF23*, *CEP131*, *MKS1*, *LRRC45*, *AURKA*, *RGS14*	0.041
GO_CC	GO:0031616~spindle pole centrosome	2	*AURKA*, *AURKB*	0.044
GO_CC	GO:0000780~condensed nuclear chromosome, centromeric region	2	*AURKA*, *AURKB*	0.044
GO_CC	GO:0005604~basement membrane	3	*LAMB2*, *SERPINF1*, *DST*	0.048
GO_CC	GO:0035371~microtubule plus-end	2	*KIF18B*, *DST*	0.073
GO_CC	GO:0051233~spindle midzone	2	*AURKA*, *AURKB*	0.081
GO_CC	GO:0005814~centriole	3	*MKS1*, *AURKA*, *PLA2G3*	0.090
GO_MF	GO:0005524~ATP binding	14	*KIF23*, *ADCY7*, *PIK3C2B*, *FLT4*, *KIF18B*, *ATP10D*, *AURKA*, *AURKB*, *UBE2B*, *MAP3K7*, *PAK2*, *DDX60*, *MLKL*, *IP6K2*	0.014
GO_MF	GO:0051015~actin filament binding	4	*AJUBA*, *CTNNAL1*, *VIL1*, *CORO6*	0.021
GO_MF	GO:0035174~histone serine kinase activity	2	*AURKA*, *AURKB*	0.022
GO_MF	GO:0070573~metallodipeptidase activity	2	*ACY1*, *PM20D1*	0.035
GO_MF	GO:0005515~protein binding	48	*CTNNAL1*, *DCBLD2*, *KIF23*, *FAM96B*, *ATP10D*, *SELENBP1*, *AURKA*, *AURKB*, *SLC19A2*, *MAP3K7*, *NRCAM*, *PEG10*, *PAK2*, *MRPL15*, *NCAPG2*, *DDX60*, *MLKL*, *CDCA3*, *IP6K2*, *CEP131*, *SSBP4*, *ACY1*, *NOS1AP*, *TONSL*, *PIK3C2B*, *FLT4*, *VIL1*, *CDHR2*, *CORO6*, *LRRC45*, *KIF18B*, *RBBP7*, *UBE2B*, *RGS14*, *STAT2*, *AJUBA*, *MKS1*, *CCNB3*, *RNF115*, *TIMELESS*, *SERPINF1*, *SERPINF2*, *CD82*, *NGFR*, *VAMP2*, *DST*, *KPNA2*, *KLF4*	0.041
GO_MF	GO:0004672~protein kinase activity	5	*MAP3K7*, *PAK2*, *AURKA*, *MLKL*, *AURKB*	0.076
GO_MF	GO:0019901~protein kinase binding	5	*CCNB3*, *PAK2*, *AURKA*, *MLKL*, *RGS14*	0.086
KEGG	hsa04015:Rap1 signaling pathway	4	*ADCY7*, *FLT4*, *NGFR*, *RGS14*	0.073
KEGG	hsa04014:Ras signaling pathway	4	*PAK2*, *FLT4*, *NGFR*, *PLA2G3*	0.086
KEGG	hsa00220:Arginine biosynthesis	2	*ACY1*, *ARG2*	0.089

**Table 2:** The predicted targets of lncRNAs.

lncRNA Name	Neighboring Genes	Coexpressed Genes	*p*-Value	Correlation	FDR	Human Syntenic lncRNA
*LOC106505099*	*HoxB2*	*NA*				*HoxB-AS1*
	*HoxB1*					
	*HoxB3*					
	*HoxB5*					
	*HoxB6*					
	*HoxB7*					
	*HoxB8*					
*LOC106509609*	*ARHGEF37*	*NA*				
	*PPARGC113*					
*LOC102168077*	*SPG7*	*DNAAF3*	1.10 × 10^−6^	0.999	0.0097	
	*RPL13*					
	*CPNE7*					
	*ANKRD11*					
*LOC102158335*	*NA*	*NA*				
*LOC100525935*	*NDST3*	*PPP3CB*	7.44 × 10^−7^	0.999	0.0066	
	*PRSS12*					
*LOC102161888*	*NDUFAF2*	*CKMT1A*	7.40 × 10^−6^	−0.998	0.065	*SMIM15-AS1*
	*SMIM15*					
	*ZSWIM6*					

**Table 3:** The enriched GO terms using the lncRNA targeted mRNAs.

Category	Term	Count	*p*-Value	Genes
GO_BP	GO:0048704~embryonic skeletal system morphogenesis	7	1.91 × 10^−12^	*HOXB3*, *HOXB1*, *HOXB2*, *HOXB7*, *HOXB8*, *HOXB5*, *HOXB6*
GO_BP	GO:0009952~anterior/posterior pattern specification	7	1.71 × 10^−10^	*HOXB3*, *HOXB1*, *HOXB2*, *HOXB7*, *HOXB8*, *HOXB5*, *HOXB6*
GO_BP	GO:0007275~multicellular organism development	5	0.0020	*HOXB3*, *HOXB1*, *HOXB2*, *HOXB7*, *HOXB8*
GO_BP	GO:0021570~rhombomere 4 development	2	0.0021	*HOXB1*, *HOXB2*
GO_BP	GO:0021612~facial nerve structural organization	2	0.0096	*HOXB1*, *HOXB2*
GO_BP	GO:0006355~regulation of transcription, DNA-templated	6	0.018	*HOXB3*, *HOXB1*, *HOXB2*, *HOXB7*, *HOXB8*, *HOXB6*
GO_BP	GO:0006351~transcription, DNA-templated	6	0.050	*HOXB3*, *HOXB1*, *HOXB2*, *HOXB7*, *HOXB8*, *HOXB6*
GO_CC	GO:0005634~nucleus	10	0.056	*HOXB3*, *HOXB1*, *HOXB2*, *HOXB7*, *RPL13*, *HOXB8*, *HOXB5*, *ANKRD11*, *CPNE7*, *HOXB6*
GO_MF	GO:0043565~sequence-specific DNA binding	6	8.80 × 10^−5^	*HOXB3*, *HOXB1*, *HOXB2*, *HOXB7*, *HOXB8*, *HOXB6*
GO_MF	GO:0003700~transcription factor activity, sequence-specific DNA binding	5	0.011	*HOXB3*, *HOXB2*, *HOXB7*, *HOXB8*, *HOXB6*

## Supplementary Materials

The following are available online at https://www.mdpi.com/2076-0817/9/6/479/s1, Figure S1: Reads distribution of sequencing results. Table S1: List of primer sequences. Table S2: The mapping statistics of the sequenced reads. Table S3: List of differentially expressed genes of PCV2 infected vs. mock infected. Table S4: List of differentially expressed lncRNAs.

## Abbreviations

BLAST, basic local alignment search tool; BP, biological process; CC, cellular component; DAVID, Database for Annotation, Visualization and Integrated Discovery tool; DEG, differentially expressed gene; FDR, false discovery rate; GO, gene ontology; HOXB, homeobox B; KEGG, Kyoto Encyclopedia of Genes and Genomes; lncRNA, long noncoding RNA; MAPK, mitogen-activated protein kinase; MF, molecular function; MOI, multiplicity of infection; ORF, open reading frame; PCV2, porcine circovirus type 2; PMWS, postweaning multisystemic wasting syndrome; QC, quality control; qRT-PCR, quantitative real-time polymerase chain reaction; RIN, RNA integrity number; SEM, standard errors of the mean.
