# Supplementary figures and images for: Identification of lncRNAs Involved in PCV2 Infection of PK-15 Cells
Source: Pathogens. 2020 Jun 17;9(6):479. doi: 10.3390/pathogens9060479 (PMC7350310; doi:10.3390/pathogens9060479)

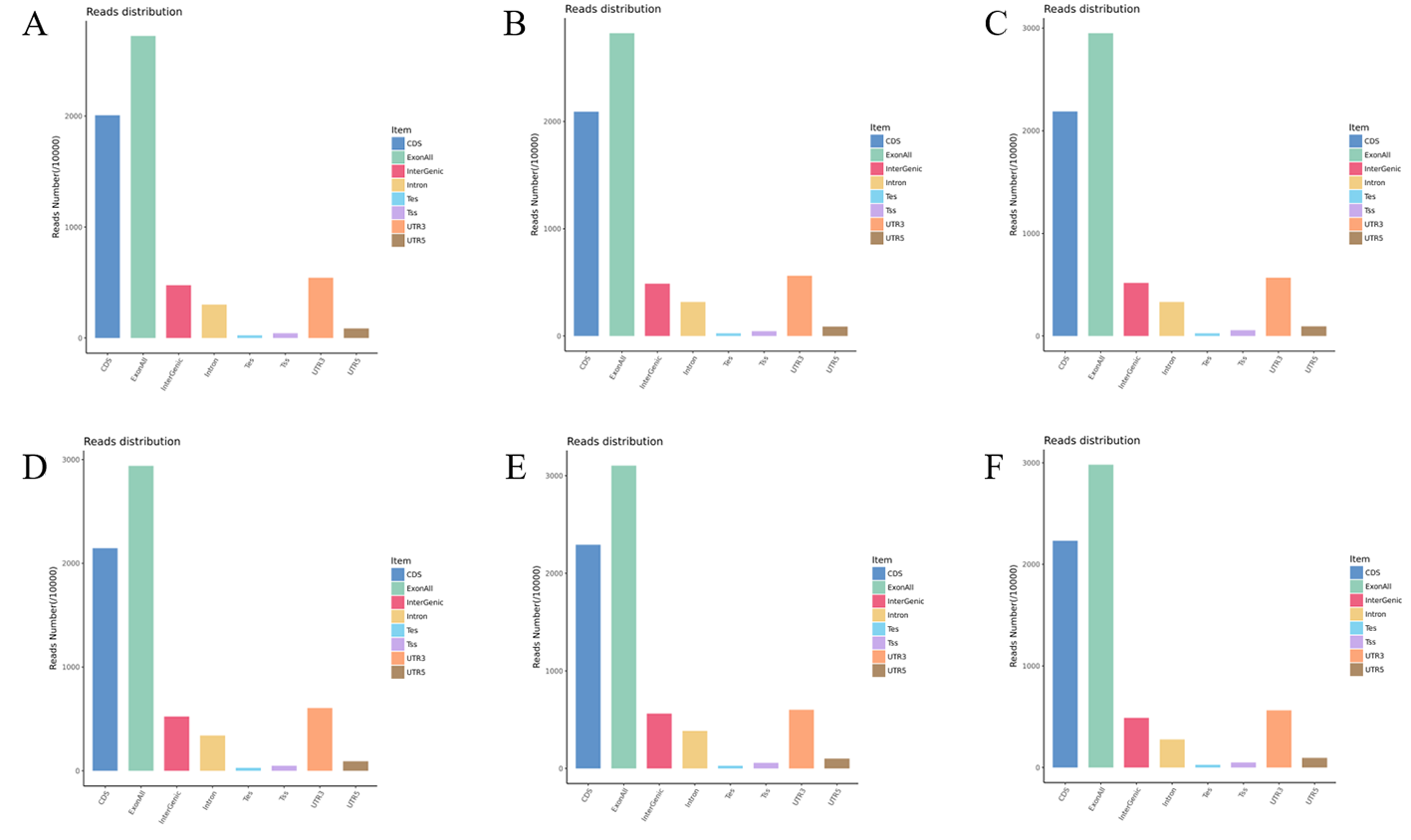

Supplement: Supplementary file 1 [file pathogens-09-00479-s001.zip › Figure S1.tif]
